# Potential antidepressant effects of Traditional Chinese botanical drug formula Chaihu-Shugan-San and its active ingredients

**DOI:** 10.3389/fphar.2024.1337876

**Published:** 2024-04-02

**Authors:** Ziyi Guo, Tianjian Long, Jianping Yao, Yamin Li, Lu Xiao, Min Chen

**Affiliations:** ^1^ Faculty of Chinese Medicine and State Key Laboratory of Quality Research in Chinese Medicines, Macau University of Science and Technology, Macau, Macao SAR, China; ^2^ Henan University of Chinese Medicine, Zhengzhou, China; ^3^ Zunyi Medical University, Zhuhai, China

**Keywords:** Chaihu-Shugan-San, depression, active ingredients, antidepressant, systematic review

## Abstract

**Background:** Depression is a severe mental disorder that poses a significant threat to both the physical and mental wellbeing of individuals. Currently, there are various methods for treating depression, including traditional Chinese herbal formulations like Chaihu-Shugan-San (CSS), which have shown effective antidepressant effects in both clinical and animal research.

**Objective:** This review aims to provide a comprehensive synthesis of evidence related to CSS, considering both preclinical and clinical studies, to uncover its potential multi-level, multi-pathway, and multi-target mechanisms for treating depression and identify its active ingredients.

**Methods:** A thorough search was conducted in electronic databases, including PubMed, MEDLINE, Web of Science, Google Scholar, CNKI, and Wanfang, using keywords such as “Chaihu Shugan” and “depression” to retrieve relevant literature on CSS and its active ingredients. The review process adhered to the PRISMA guidelines.

**Results:** This review consolidates the mechanisms underlying antidepressant effects of CSS and its active ingredients. It emphasizes its involvement in the regulation of monoaminergic neurotransmitter systems, synaptic plasticity, and the hypothalamic-pituitary-adrenal axis, among other aspects.

**Conclusion:** CSS exerts a pivotal role in treating depression through various pathways, including the monoaminergic neurotransmitter system, the hypothalamic-pituitary-adrenal axis, synaptic plasticity, inflammation, brain-derived neurotrophic factor levels, and the brain-gut axis. This review facilitates a comprehensive understanding of the current state of CSS research, fostering an in-depth exploration of the etiological mechanisms of depression and the potential discovery of novel antidepressant drugs.

## 1 Introduction

Depression has emerged as one of the most prevalent global public health issues, with its incidence steadily rising year by year. Depression patients commonly exhibit persistent low mood, insomnia, extreme fatigue, lack of energy, self-doubt, and excessive self-blame. They may experience unexplained physical symptoms, and in some cases, thoughts of self-harm or suicide may be present. Both the recurrence rate and the disability rate associated with depression are substantial (GBD, 2019 Mental Disorders Collaborators, 2022). This debilitating condition affects a staggering 350 million individuals worldwide, which continues to grow.

The economic burden of depression is substantial, with annual cost estimates reaching as high as 200 billion dollars. This accounts for the expenses associated with depression treatment and the productivity impact related to work, such as absenteeism and missed deadlines. Consequently, the societal impact of depression is becoming increasingly severe (Greenberg et al., 2015).

Projections from a study indicate that by 2030, depression is poised to become the second-largest contributor to the global disease burden (Mathers and Loncar, 2006). The likelihood of severe depression is significantly higher among individuals with common physical illnesses such as cardiovascular diseases, cancer, and neurodegenerative diseases compared to the general population. Conversely, individuals with depression are at a greater risk of developing various physical illnesses like cardiovascular diseases (Basiri et al., 2023), stroke (Ji et al., 2023), and diabetes (Qi et al., 2023). This high level of comorbidity is associated with worse outcomes, reduced treatment compliance, increased mortality rates, higher healthcare utilization, and costs. Comorbidities can also lead to a range of clinical challenges, including more complex treatment regimens, issues related to adaptive health behaviors, drug interactions, and adverse events caused by medications used for both physical and mental disorders (Berk et al., 2023). There are numerous risk factors associated with the onset of depression, including stress, pain, physical illnesses, and cognitive decline. Currently, scholars are primarily focusing on the pathophysiology of depression, particularly in relation to the monoaminergic system, the glutamatergic system, the hypothalamic-pituitary-adrenal axis, inflammation, gut microbiota, and neurogenesis (Emiko Tsugiyama et al., 2023; Reyes-Lizaola et al., 2023). Since the 1950s, commonly used clinical antidepressant medications have primarily targeted the increase of serotonin or acted directly on serotonin receptors (Murrough and Charney, 2012; Undurraga and Baldessarini, 2012). Although 35%–50% of patients do not respond to these medications, selective serotonin reuptake inhibitors (SSRIs) continue to be the mainstay of antidepressant therapy. Additionally, drowsiness and gastrointestinal reactions are common adverse effects of these drugs. Improvement in depressive symptoms typically takes at least 2 weeks or more after starting treatment (Trivedi et al., 2006; Trivedi et al., 2008). Reportedly, traditional Chinese medicine has demonstrated high efficacy in the treatment of depression with minimal side effects, making it considered a favorable approach for managing depressive symptoms. A substantial body of research indicates that traditional Chinese medicine may significantly alleviate the symptoms of depression and other disorders characterized by depressive behaviors (Moreira et al., 2023).

**FIGURE 1:**
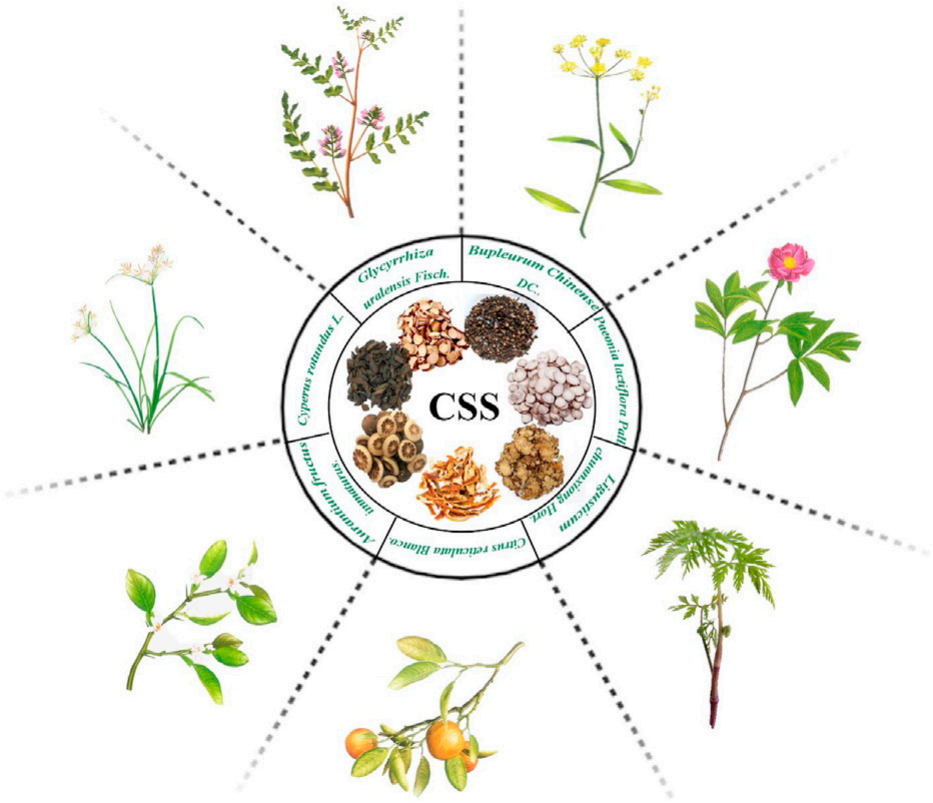
The seven botanical drugs used in Chaihu-Shugan-San.

Chaihu-Shugan-San (CSS) is a traditional Chinese herbal formula that was originally documented in the Ming Dynasty’s “Yi Xue Tong Zhi.” It comprises seven botanical drugs, including Bupleurum chinense DC. [Apiaceae; Bupleuri radix], Citrus reticulata Blanco [Rutaceae; Citri reticulatae pericarpium], Paeonia lactiflora Pall. [Paeoniaceae; Paeoniae radix alba], Cirtus aurantium L. [Rutaceae; Aurantii fructus], Cyperus rotundus L. [Cyperaceae; Cyperi rhizoma], Ligusticum chuanxiong Hort. [Apiaceae; Chuanxiong rhizoma], and Glycyrrhiza uralensis Fisch. [Fabaceae; Glycyrrhizae radix et rhizoma], and the ratio is 4:4:3:3:3:3:1 (Li et al., 2022). The specific preparation method involves mixing 7 types of botanical drugs in the respective proportions, soaking them in water at 25°C for 0.5 h, and then heating to 100°C and boiling for 30 min. The first filtrate is collected in a beaker. The botanical drug residues are refluxed and heated in the same volume of water for 30 min, and then the second filtrate is collected. The two filtrates are integrated and filtered through 5 layers of cotton gauze (Yan et al., 2020; Zhu et al., 2021). All plant names or species were validated using http://mpns.kew.org/mpns-portal/ and Chinese Pharmacopoeia, as depicted in Figure 1. CSS has been long used in clinical practice for the treatment of liver qi stagnation combined with depression. Clinical research has shown that CSS exhibits significant efficacy and safety in alleviating depressive symptoms in patients, leading to improvements in depression symptomatology, increased response rates (Wang et al., 2012), and enhanced HAMD scores (Ding et al., 2018). Moreover, CSS has demonstrated positive antidepressant effects in various populations, such as *postpartum* depression, perimenopausal depression (Cai and Bao, 2023), post-stroke depression (Fan et al., 2023), and geriatric depression (Xiong, 2021).

Additionally, research suggests that CSS not only displays antidepressant effects in common rodent models of depression, including chronic unpredictable mild stress (CUMS) (Liu et al., 2018) or combined social isolation (Zhang et al., 2011a), chronic restraint stress (CRS) (Qian et al., 2020) or combined social isolation (Lijuan et al., 2012), and LPS-induced depression model (Xuesong et al., 2023).

Prior research has identified several potential antidepressant mechanisms of CSS. These mechanisms include the regulation of monoaminergic neurotransmitters (Hwang et al., 2020), effects on the HPA axis, anti-inflammatory and neuroplasticity actions (Li et al., 2019), primarily affecting regions such as the hippocampus (Zhu et al., 2021), prefrontal cortex (Zhang et al., 2021a) and amygdala (Ying et al., 2011). Extensive research employing various techniques and methods has been conducted to investigate the antidepressant mechanisms of CSS and its active ingredients. However, due to certain limitations, including its complex composition, the exact antidepressant mechanisms of CSS remain to be fully elucidated. Therefore, this paper provides a comprehensive review of its antidepressant mechanisms and active ingredients, with the hope that our findings will contribute to further research on CSS in the context of depression.

## 2 Methods

### 2.1 Search strategy

We searched for CSS about depressive disorders using the terms described below to obtain data from several electronic databases from the inception of each database to October 2023. Specifically, we searched using PubMed, Web of Science, Google Scholar, Cochrane Library, Chinese National Knowledge Infrastructure, VIP Information, and Wanfang Database. We used the search strategy of combining words (“Depression” OR “Depressive Symptoms” OR “Symptom, Depressive”) AND (“Chaihushugansan” OR “Chai Hu Shu Gan San” OR “Chai-Hu-Shu-Gan-San” OR “Chaihu Shugan San” OR “Chai Hu Shu Gan formula” OR “Chai Hu Shu Gan powder”).

### 2.2 Inclusion and exclusion criteria

The inclusion criteria were defined as follows: 1) Studies, both *in vivo* and *in vitro*, that evaluated the effects of CSS in the treatment of depression; 2) Any intervention in the experimental group that involved the use of an CSS prescription for depression, comprising the seven botanical drugs mentioned earlier, at any dosage, frequency, or administration method; 3) No restrictions on the species, sex, age, or weight of animals, and the species of cells under investigation; and 4) Studies that focused on depression. The exclusion criteria were as follows: 1) The use of CSS as an adjuvant drug in the intervention group; 2) Duplicate studies; 3) Inadequate outcome measures or incomplete data in the studies.

## 3 Clinical studies on Chaihu-Shugan-San

### 3.1 Clinical research and safety of CSS for depression

Multiple meta-analyses have demonstrated that when CSS is used in conjunction with antidepressant drugs such as fluoxetine, venlafaxine, paroxetine, etc., the efficacy in treating depression significantly surpasses that of using antidepressants alone, indicating that the combination of CSS with antidepressants effectively enhances the treatment outcomes (Zeng et al., 2017; Sun et al., 2020). In a study involving 40 *postpartum* depression patients who received 4 weeks of CSS treatment, it was found that the patients experienced a reduction in their *postpartum* depression scores, as measured by the Edinburgh *Postartum* Depression Scale (EPDS) and the Hamilton Depression Scale (HAMD). Simultaneously, their hormone levels, including luteotropic hormone (LH), estradiol (E2), and follicular stimulating hormone (FSH), increased. This suggests that CSS can effectively ameliorate clinical symptoms of depression and regulate hormone levels. In a clinical study involving 120 geriatric depression patients, after CSS treatment, it was observed that HAMD scores, Pittsburgh Sleep Quality Index (PSQI) scores, and Quality of Life (QOL) scores all improved. This indicates that CSS can effectively reduce the severity of depression, improve sleep quality and life quality of patients (Xiong et al., 2019).

When CSS was used as the primary treatment, a network meta-analysis of seven traditional Chinese medicines as adjunctive therapy for post-stroke depression revealed that CSS has unique advantages in enhancing clinical efficacy compared to some other traditional Chinese medicines (Yu et al., 2022). In a clinical randomized controlled trial involving 86 patients, CSS demonstrated a significantly higher clinical effectiveness rate compared to the control group (97.67% vs. 81.40%) and substantially alleviated depression levels of patients (Zhang, 2016). Results from a clinical study indicated that, when used as an adjunctive medication, clinical effectiveness of CSS (overall efficacy rate of 97.20%) was significantly better than the control group receiving only psychotherapy (overall efficacy rate of 91.42%) (Chen et al., 2021).

### 3.2 Clinical application of CSS in the treatment of depression

In a systematic review and meta-analysis assessing the safety and efficacy of CSS in treating depression, it was observed that the adverse reaction rate of CSS combined with antidepressants was lower than that of the group receiving antidepressants alone (Zeng et al., 2017; Sun et al., 2020). An analysis of a randomized controlled trial involving 66 patients treated with CSS for post-stroke depression showed that CSS significantly improved scores on related scales, including the Hamilton Anxiety Rating Scale, and that CSS in combination with antidepressants effectively treated post-stroke depression with lower side effects compared to the control group (6.06% vs. 27.27%) (Tao et al., 2023). These studies suggest that the likelihood of CSS interacting with other medications is quite low.

However, further investigation is needed to assess the safety of using CSS alone. In a meta-analysis involving CSS for Parkinson’s disease patients, data from 11 trials showed no adverse reactions, indicating that CSS could be a potential treatment option for patients with comorbid depression (Huber et al., 2023). A meta-analysis of 24 studies using CSS or its modified formulas for treating depression reported neurological symptoms such as dizziness, headache, sleep disturbances, hyperactivity, blurred vision, and fatigue, as well as gastrointestinal symptoms including dry mouth, diarrhea, anorexia, nausea, vomiting, and constipation. However, the incidence of adverse reactions was lower than in the control group (Zhao et al., 2023b).

## 4 The antidepressant mechanisms of CSS

### 4.1 Regulation of monoaminergic neurotransmitter systems

**FIGURE 2:**
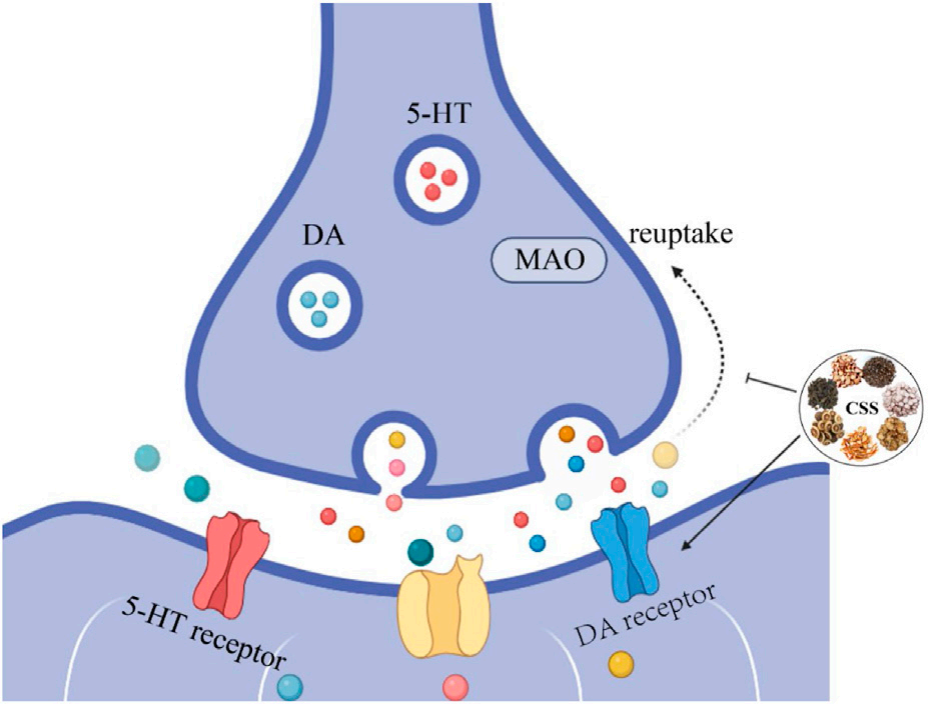
CSS Increases Monoaminergic Neurotransmitter Levels. CSS facilitates the synthesis of monoaminergic neurotransmitters, leading to an increase in the concentration of these neurotransmitters. Inhibiting the reuptake of monoaminergic neurotransmitters enhances their respective signaling pathways, elevates monoaminergic neurotransmitter levels, and alleviates depressive symptoms.

Damage to the monoaminergic neurotransmission and the accompanying decrease in 5-HT and NE concentrations are major factors in the pathogenesis of depression and are the targets of most antidepressants (Perez-Caballero et al., 2019). Currently, most antidepressants work by inhibiting the reuptake of monoamines, thereby increasing monoamine levels in the synaptic cleft (Lee et al., 2010). However, selective serotonin reuptake inhibitors may lead to withdrawal symptoms such as nausea, vomiting, and diarrhea, limiting their clinical use (Lee et al., 2010). As shown in Figure 2, After treatment with CSS, the content of 5-HT, 5-HIAA, and DA in the hippocampus and hypothalamus of rats increased, while the NE content decreased. This suggests that CSS exerts its antidepressant effects by regulating levels of monoaminergic neurotransmitters like 5-HT and DA in the rat brain (Yufeng et al., 2016). Furthermore, earlier research has also yielded the same conclusion (Wang et al., 2014). MAO-A is involved in the metabolism of 5-HT and NE, and some antidepressants can exert their effects by inhibiting MAO activity (Jahanabadi et al., 2023). Studies have shown that CSS effectively reduces the activity of MAO enzymes, regulating the expression of 5-HT and NE, thereby achieving its antidepressant effects (Haiying et al., 2011).

Tryptophan (Trp) is an essential amino acid taken in by the human body from external sources. It serves as the primary metabolic precursor for the production of serotonin (5-HT) and kynurenine (KYN), which regulate various metabolic activities (Zádori et al., 2018). Under normal physiological conditions, the activity of indoleamine-2,3-dioxygenase (IDO) is low, and Trp is primarily metabolized by tryptophan-2,3-dioxygenase (TDO) in the liver. However, in the presence of inflammation or stress, proinflammatory cytokines can induce a significant increase in IDO activity, and elevated glucocorticoids further activate TDO, directing Trp toward the kynurenine pathway (KP), resulting in a decrease in central and peripheral 5-HT levels (Murakami et al., 2016). In normal mammals, approximately 40% of brain KYN comes from central metabolism, while the remaining 60% originates from the periphery. Therefore, peripheral KYN levels may to some extent represent the levels of central KP metabolites (Li et al., 2020a). In 72 untreated depressive patients, it was found that the levels of KP metabolites in cerebrospinal fluid were closely correlated with those in plasma, with the KYN/Trp ratio being the most correlated indicator (Haroon et al., 2020). In mice after treatment with CSS, the ratio of 5-HT/TRP in the liver significantly increased, while the ratio of KYN/TRP in tissues such as the liver, colon, and brain significantly decreased (Mengyu et al., 2021). Additionally, CSS reduced the activity and expression levels of TDO in the liver, leading to an increase in mouse 5-HT expression levels and the alleviation of depression-like behavior.

### 4.2 Maintaining homeostasis in the regulated HPA axis

One prominent feature of depression is the dysfunction of the hypothalamic-pituitary-adrenal (HPA) axis (Menke, 2023). HPA axis hyperactivity is particularly common in individuals with depression and can affect their cognitive functions (Cherian et al., 2019). Excessive stress stimuli to the brain result in cortical impact, triggering the release of signals from the hypothalamus. This leads to increased secretion of corticotropin-releasing factor (CRF) in the HPA axis. Excess CRF is transported through the portal venous system to the pituitary, where it stimulates the synthesis of adrenocorticotropic hormone (ACTH). This, in turn, promotes the production of cortisol (CORT) by the adrenal cortex (Dwyer et al., 2020). CRF is a key regulator of the body’s stress response and is closely associated with various psychiatric disorders, including depression. Overproduction of CRF is a mechanism that activates the HPA axis (Montgomery et al., 2023). Furthermore, cortisol (CORT), which acts downstream of the HPA axis, plays a significant role in the pathogenesis of depression. The CORT levels in individuals with depression are closely related to their clinical symptoms (Holloway et al., 2023). In a study involving perimenopausal depression rats treated with CSS for 21 days, both CRH and CORT levels significantly increased, indicating that CSS can regulate CRH and CORT levels to restore HPA axis function (Shengqiang et al., 2015). Similarly, in the case of CUMS combined with social isolation-induced depression in rats, CSS exhibited the same antidepressant effects (Yunhui et al., 2009).

**FIGURE 3:**
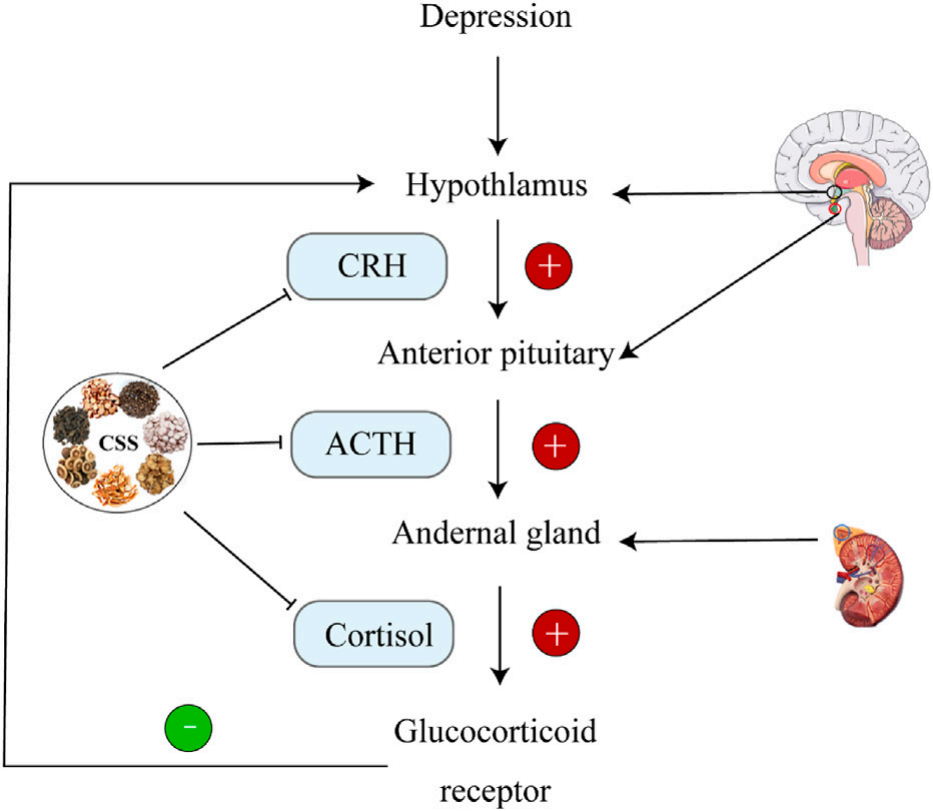
CSS Regulates HPA Axis Homeostasis. CSS maintains HPA axis homeostasis by inhibiting ACTH, CRH, and CORT.

During stress, corticotropin-releasing hormone (CRH) secreted by the paraventricular nucleus (PVN) of the hypothalamus stimulates the secretion of ACTH and glucocorticoids (GC); corticosterone in rodents (Kim et al., 2019). Receptors for glucocorticoids primarily include mineralocorticoid receptors (MR) and glucocorticoid receptors (GR). During stress, the expression of GC increases dramatically. After crossing the blood-brain barrier, GC acts on hippocampal GR. The GC-GR complex formed in response to high GC concentrations inhibits CRH transcription in the hypothalamus, providing negative feedback regulation on the HPA axis, thus suppressing HPA axis hyperactivity (de Kloet and Joëls, 2023). GC levels are crucial indicators for evaluating HPA axis function. The hippocampus, as the central regulator of the HPA axis, is rich in GR and mineralocorticoid receptors (MR). It is highly susceptible to the influence of high concentrations of glucocorticoids during stress (Gong et al., 2023). As illustrated in Figure 3, when CSS was orally administered to CUMS rats continuously for 21 days, it was found that CRH levels increased and GR levels decreased in the hippocampus compared to the depression model group. This indicates that CSS can effectively weaken the negative feedback effect of GC on the HPA axis, alleviating HPA axis hyperactivity. Thus, CSS may regulate HPA axis hyperactivity by acting on the hippocampus to achieve its antidepressant effect (Fan et al., 2015).

### 4.3 Upregulation of BDNF levels

Brain-derived neurotrophic factor (BDNF) is a crucial member of the neurotrophic factor family, initially discovered in pig brain in the 1980s (Leibrock et al., 1989). BDNF is primarily synthesized in neurons and is distributed throughout the central nervous system (CNS). It plays a role in synaptic plasticity recovery, 5-HT signal transduction, and regulating the levels of 5-HT in the brain (Bhattarai et al., 2020; Costa et al., 2022). Numerous studies have shown that BDNF is closely associated with the occurrence, development, and treatment of depression. In the field of neurobiology, BDNF is one of the most studied neurotrophic factors (Amagase et al., 2023; Wang et al., 2023a). Changes in BDNF activity and levels in the brain are closely related to the development of depression. Autopsy results have revealed that the expression levels of BDNF in the plasma of depressed patients are lower than in the control group (Gadad et al., 2021). Furthermore, research suggests that developing antidepressants targeting BDNF might be one of the most effective strategies for future antidepressant drug development (Deltheil et al., 2009; Furukawa et al., 2019).

**FIGURE 4:**
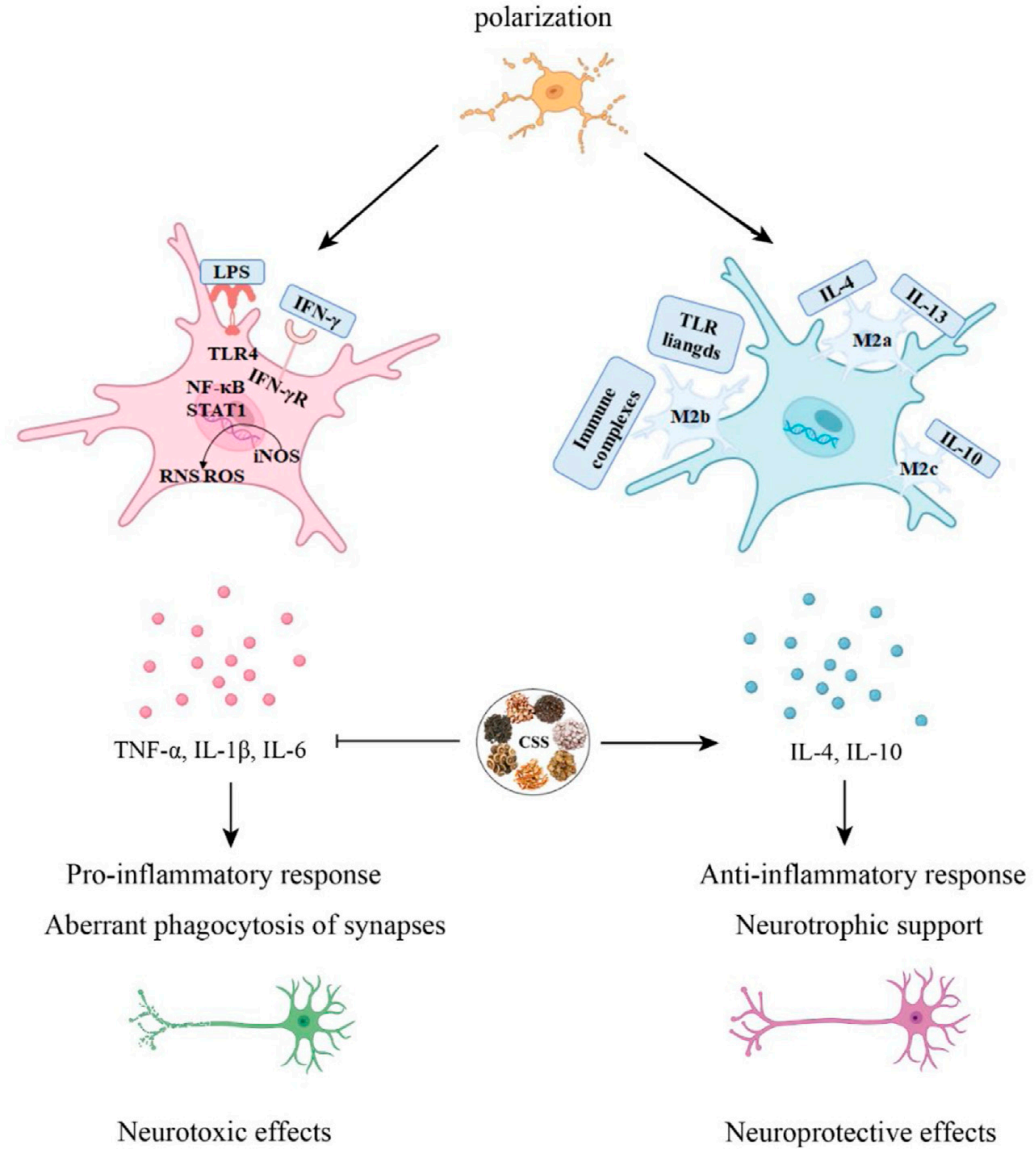
CSS alleviates neuroinflammation. Microglia can differentiate into M1 and M2 phenotypes. CSS is capable of exerting neuroprotective effects by modulating the immune response through the regulation of pro-inflammatory and anti-inflammatory cytokines in microglial activation.

In a study involving CUMS rats subjected to 28 days of CSS oral administration, it was observed that compared to the depression model group, the expression of BDNF in the prefrontal cortex of rats significantly increased. This indicates that CSS can exert effects similar to the antidepressant venlafaxine by regulating BDNF expression levels (Zhang and Liu, 2020). Tropomyosin receptor kinase B (TrkB) is a specific receptor for BDNF, and it has been confirmed that activating BDNF-TrkB signaling can have an antidepressant effect (Ma et al., 2020). Figure 4 After intervening with CSS in depression model rats, it was found that compared to the model group, rats in the CSS group exhibited significantly increased expression of BDNF and its receptor TrkB in the hippocampus, prefrontal cortex, and amygdala, suggesting that the mechanism through which CSS alleviates depressive states might be related to the increased expression of BDNF and its receptor TrkB in these brain regions (Ying et al., 2011).

### 4.4 Promoting synaptic plasticity

**FIGURE 5:**
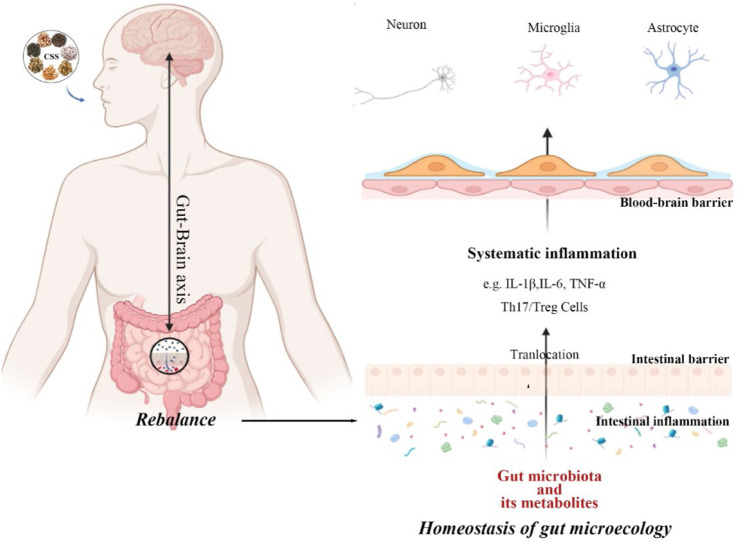
CSS restores dysbiosis of the gut microbiota. CSS exerts a protective effect on neuronal cells by regulating the gut microbiota through the brain-gut axis, activating the systemic immune response, and crossing the blood-brain barrier.

Synapses are the fundamental structural units responsible for transmitting information in neurons. Changes in their morphology, quantity, density, and signal transmission efficiency are collectively referred to as synaptic plasticity (Suvrathan et al., 2016; Sweatt, 2016). Synaptic plasticity serves as the neurobiological foundation for the growth and development of the nervous system, repair after injury, and the processes of learning and memory (Sehgal et al., 2013). Figure 5 When examining synaptic structural changes under electron microscopy, it is observed that in comparison to the model group, synaptic improvements occur in the hippocampal region of CUMS-depressed rats following CSS intervention, with an increase in synaptic quantity. This suggests that CSS can effectively enhance synaptic plasticity in the hippocampal region of depressed rats (Fan et al., 2018).

Two specific protein markers, PSD95 and SYN, are used to measure synaptic plasticity. PSD95 is abundantly expressed in the postsynaptic density region and interacts with membrane receptors, ion channels, and cell adhesion molecules, thereby participating in the regulation of synaptic plasticity and learning and memory abilities. SYN is a crucial presynaptic vesicle membrane protein, and its expression accurately reflects synaptic distribution, quantity, and density, making it the most direct indicator of changes in synaptic plasticity. Research indicates that the continuous administration of CSS through oral gavage to depressed rats for 2 weeks results in a significant increase in the deposition of Syn-positive substances in the CSS group compared to the model group. This suggests that CSS can effectively inhibit hippocampal neuronal apoptosis, impact the expression of SYN and PSD95, promote the recovery of damaged synaptic structure and function, and consequently ameliorate depressive-like behaviors in rats (Lan et al., 2023).

NMDAR (N-methyl-D-aspartate receptor) is a glutamate-gated ion channel that regulates neuronal survival, dendritic and axonal development in neurons, excitatory synaptic plasticity, and the formation of neuronal circuits (Tashiro et al., 2006). NMDAR is considered a necessary condition for dendritic spine structural remodeling and is believed to be the cellular basis of learning and memory (Matsuzaki et al., 2004). Downregulation of hippocampal NR2A/NR2B has been associated with cognitive impairment (Luciano-Jaramillo et al., 2019). According to the NMDAR signaling pathway, TrkB is rapidly and persistently activated, serving as a crucial binding partner for BDNF in various forms of synaptic plasticity. Studies indicate that the increase in BDNF exerts neuroprotective effects by transducing the NR2A-CaMKIV-TORC1 pathway (Xu et al., 2015). CSS enhances the expression of BDNF, NR2A, and NR2B in the hippocampus, suggesting that the antidepressant effects of CSS may be mediated through the transduction of the NR2A-CaMKIV-TORC1 pathway, impacting hippocampal neuronal development and thus restoring the neural function of damaged synapses (Xu et al., 2021).

### 4.5 Alleviating neuroinflammation

Neuroinflammation is an innate immune response of the nervous system (Meseguer et al., 2014), and it plays a role in the pathological processes of various neurological and psychiatric disorders. Patients with severe depression and animal models of depression often exhibit elevated levels of pro-inflammatory cytokines such as IL-1β, IL-6, and TNF-α (Yang et al., 2023). Cytokines are secreted proteins with growth, differentiation, and activation functions. They regulate and determine the nature of immune responses, control immune cell trafficking, and organize the cellular arrangement of immune organs (Borish and Steinke, 2003). In the context of neurodevelopment, the role of cytokines is reflected in pro-inflammatory cytokines impairing neurogenesis, while anti-inflammatory cytokines protect or promote neurogenesis (Soares et al., 2023).

Neuroglial cells play a significant role in the normal physiological processes of the central nervous system, including synaptic transmission, neural plasticity, regulation of neurons, and the local microenvironment (Fang et al., 2023). Microglial cells are the resident immune cells of the CNS, constituting approximately 5%–10% of CNS cells in mice and 0.5%–16.6% in human CNS cells. They participate in pathogen clearance, phagocytosis of dead or apoptotic cells, and the promotion or inhibition of inflammation to maintain brain health. Under normal physiological conditions, microglial cell bodies have elongated processes and continually monitor changes in the brain environment. However, in response to pathological stimuli, microglia are rapidly activated, their numbers increase, and their cell bodies enlarge while their processes become shorter (Zhu et al., 2023). Under pathological conditions, microglia transition from a resting state to an activated state, adopting two polarization phenotypes, M1 and M2. M1 microglial cells secrete many inflammatory mediators to engulf pathogens, while M2 microglial cells secrete protective cell regulators and participate in repairing local tissues and the microenvironment, thus protecting neural tissue (Smith et al., 2012; Pozzo et al., 2019). In an *in vitro* post-stroke depression model, microglial cell polarization shifts toward the M1 phenotype. However, treatment with GSK3β overexpression virus, CSS, or JAK-STAT3 inhibitors can reverse this polarization. Analysis of inflammation-related protein levels and expression of the JAK/STAT3-GSK3β/PTEN/Akt pathway reveals that the antidepressant effects of CSS may be achieved through the activation of the JAK/STAT3-GSK3β/PTEN/Akt pathway, regulating microglial cell polarization and suggesting that CSS exerts its antidepressant effects by inhibiting neuroinflammation (Fan et al., 2023). CSS can inhibit neuroinflammation in the brain by activating the important SIRT1/NF-κB inflammation signaling pathway in the prefrontal cortex of rats, thus exhibiting its antidepressant effects (Di and Liu, 2021). CSS can reduce the levels of serum TNF-α and IL-6 in depressed rats. It can also effectively inhibit the activation of hippocampal p38 mitogen-activated protein kinase (p38MAPK) and activate the extracellular signal-regulated kinase 5 (ERK5) signaling pathway to achieve its antidepressant effects (Qiu, 2014).

### 4.6 Regulation of biological activities within gut

Research has shown that the composition of the gut microbiota is altered in individuals with depression compared to healthy individuals, particularly in terms of microbial diversity and the relative abundance of specific bacterial taxa. This suggests a connection between gut dysbiosis and depression. In depression, the phyla Firmicutes, Actinobacteria, and Bacteroidetes are the most affected (Nikolova et al., 2021). After 28 days of gastric gavage treatment with CSS in CUMS, fresh fecal samples were collected and analyzed. It was observed that in the CSS group, the abundance of Firmicutes significantly increased, and the abundance of Actinobacteria decreased markedly compared to the model group. This indicates that CSS can regulate gut microbiota at the phylum level, mitigating the dysbiosis caused by chronic stress (Meng et al., 2020). In CRS mice, CSS intervention led to an increase in the relative abundance of Actinobacteria. While the results differ, a study that performed 16s rRNA sequencing of gut microbiota in patients with depression found a significant disruption in gut microbiota composition (Han et al., 2021). Differences in bacterial strain abundance led to a reduced ability of MDD patients to produce short-chain fatty acids (SCFA). The lack of SCFAs may weaken gut barrier function (Zheng et al., 2016). Metabolites from the gut, microbial cell components, and even the gut microbiota itself translocate systemically through a compromised gut barrier, often referred to as “leaky gut.” This exacerbates systemic inflammation, such as Th17/Treg imbalance, IL-6, IL-1β, and TNF-α, all of which are closely related to the pathogenesis of depression. Further induced immune responses may be related to the onset and development of depression (Młynarska et al., 2022). Therefore, the antidepressant effect of CSS may be achieved through the regulation of gut microbiota, reshaping the structure and composition of the gut microbiota, and exerting its effects through the microbiota-gut-brain axis (Ma et al., 2022).

### 4.7 System biology research

Traditional Chinese botanical drug formulations are characterized by complex compositions, making it challenging to characterize functional biomarker ingredients and understand the intricate compatibility rules of multiple ingredients. Research indicates that employing multi-omics approaches is more advantageous for identifying critical ingredients in traditional Chinese medicine (TCM), which is approach helps identify systemic targets for diseases and facilitate a deeper exploration of the mechanisms behind TCM formulations (Wang et al., 2021c). Commonly used methods in this context include network pharmacology, proteomics, and metabolomics.

Network pharmacology analysis has revealed 24 potential targets for the treatment of depression by CSS. Functional enrichment analysis indicates that the PI3K/AKT signaling pathway may be significantly influenced by CSS in MDD treatment. *In vivo* experiments demonstrate that CSS can ameliorate depressive-like behaviors in CUMS mice and promote neurogenesis. Additionally, CSS can increase the phosphorylation of PI3K/PI3K and AKT/AKT levels while reducing GSK3β/GSK3β levels in the hippocampus of CUMS mice (Zhang et al., 2021b). Research utilizing combined network pharmacology and bioinformatics analysis has identified that the mechanism of CSS primarily focuses on two main cascading signal modules. The first module involves a cascade reaction through ADCYAP1-ADCYAP2R3-GNAS-ADCY3-cAMP-PRKACA, controlling downstream genes like GRIA2, GRIN8A, GSK1A, CREB1, BDNF, FOS, ATF1, MAPK1, and JUND, which play a role in treating depression. The second module targets FOS for depression treatment through the DRD1/5-GNAQ-PLC B1-DAG-PRKCA cascade signal (Wang et al., 2021b).

Using quantitative proteomics and bioinformatics analysis methods to identify and analyze CSS, it appears that CSS may exert its antidepressant effects by influencing the synthesis, release, reuptake, and degradation pathways of GABA. Proteins such as Gad2, Vamp2, and Pde2a may be associated with the alleviation of depression by CSS. Therefore, these proteins may serve as potential antidepressant targets for CSS. Previous studies have demonstrated that CSS exerts its antidepressant effects by modulating the HPA axis and inhibiting neurotransmitter reuptake, such as norepinephrine, serotonin, and dopamine (Zhang et al., 2011b). Hence, it is hypothesized that CSS affects the glutamate and GABA signaling pathways via the HPA axis for depression treatment (Zhu et al., 2021) Table 1.

**TABLE 1:** Clinical research of CSS.

Treatment	Dosage form	Control	Treatment time	Sample (T/C)	Diagnostic criteria	Outcome index	References
Citalopram (20 mg/d) +CSS (Bupleuri radix 15g, Citri reticulatae pericarpium 6g, Paeoniae radix alba 15g, Aurantii fructus 10g, Cyperi rhizome 10g, Chuanxiong rhizome 10 g, Glycyrrhizae radix et rhizome 6g, 1dose/d)	decoction	Citalopram (20 mg/d)	8 weeks	40/39	CCMD-3	HAMD, PSQI, SF-36	Li et al. (2022)
Sertraline (50–100 mg/d) +CSS (Bupleuri radix 15g, Citri reticulatae pericarpium 15g, Paeoniae radix alba 15g, Aurantii fructus 12g, Cyperi rhizome 10g, Chuanxiong rhizome 10 g, Glycyrrhizae radix et rhizome 12g, 1dose/d)	decoction	Sertraline (50–100 mg/d)	4 weeks	68/68	ICD-10	HAMD, HAMA	Zhang et al. (2021a)
Fluoxetine (20 mg/d)+CSS (Bupleuri radix 8g, Citri reticulatae pericarpium 10g, Paeoniae radix alba 5g, Aurantii fructus 10g, Cyperi rhizome 5g, Chuanxiong rhizome 10 g, Glycyrrhizae radix et rhizome 10g, 1dose/d)	decoction	Fluoxetine (20 mg/d)	8 weeks	41/42	CCMD-3	HAMD, HAMA	Zhao et al. (2023a)
Venlafaxine (5 mg/d) +CSS (Bupleuri radix 9g, Citri reticulatae pericarpium 12g, Paeoniae radix alba 12g, Aurantii fructus 6g, Cyperi rhizome 6g, Chuanxiong rhizome 6 g, Glycyrrhizae radix et rhizome 3g, 1dose/d)	decoction	Venlafaxine (5 mg/d)	4 weeks	30/30	ICD-10	HAMD	Zhu et al. (2021)
Amitriptyline (25 mg/d)+ CSS (Bupleuri radix 15g, Citri reticulatae pericarpium 15g, Paeoniae radix alba 15g, Aurantii fructus 12g, Cyperi rhizome 15g, Chuanxiong rhizome 15 g, Glycyrrhizae radix et rhizome 3g, 1dose/d)	decoction	Amitriptyline (25 mg/d)	12 weeks	(35/34)	CCMD-3	HAMD, SDS,SAS	Liu et al. (2017)

Serum metabolomics results indicate that 16 proteins and 63 genes are regulated by CSS. Sphingolipid metabolism and arachidonic acid metabolism appear to be the most significantly affected metabolic pathways related to depression induced by CUMS. Multiple studies have demonstrated that depression often accompanies sphingolipid metabolism disturbances (Li et al., 2022), and clinically, low levels of arachidonic acid in blood are associated with adult depression (Tsuchimine et al., 2015). Metabolomic studies of the hippocampus and serum in chronic variable stress (CVS)-induced rats reveal 10 metabolites from the hippocampus and 11 from serum that are considered potential biomarkers involved in the development of depression. CSS collaboratively regulates metabolic network abnormalities, including energy metabolism, neurotransmitter synthesis, tryptophan, phospholipids, fatty acids, bile acid metabolism, bone loss, and liver detoxification. CSS reverses the decrease in BDNF, ERK2/1, and pERK2 in CVS rats, suggesting that the ERK signaling system may be one of the targets for antidepressant effects of CSS (Su et al., 2014).

**FIGURE 6:**
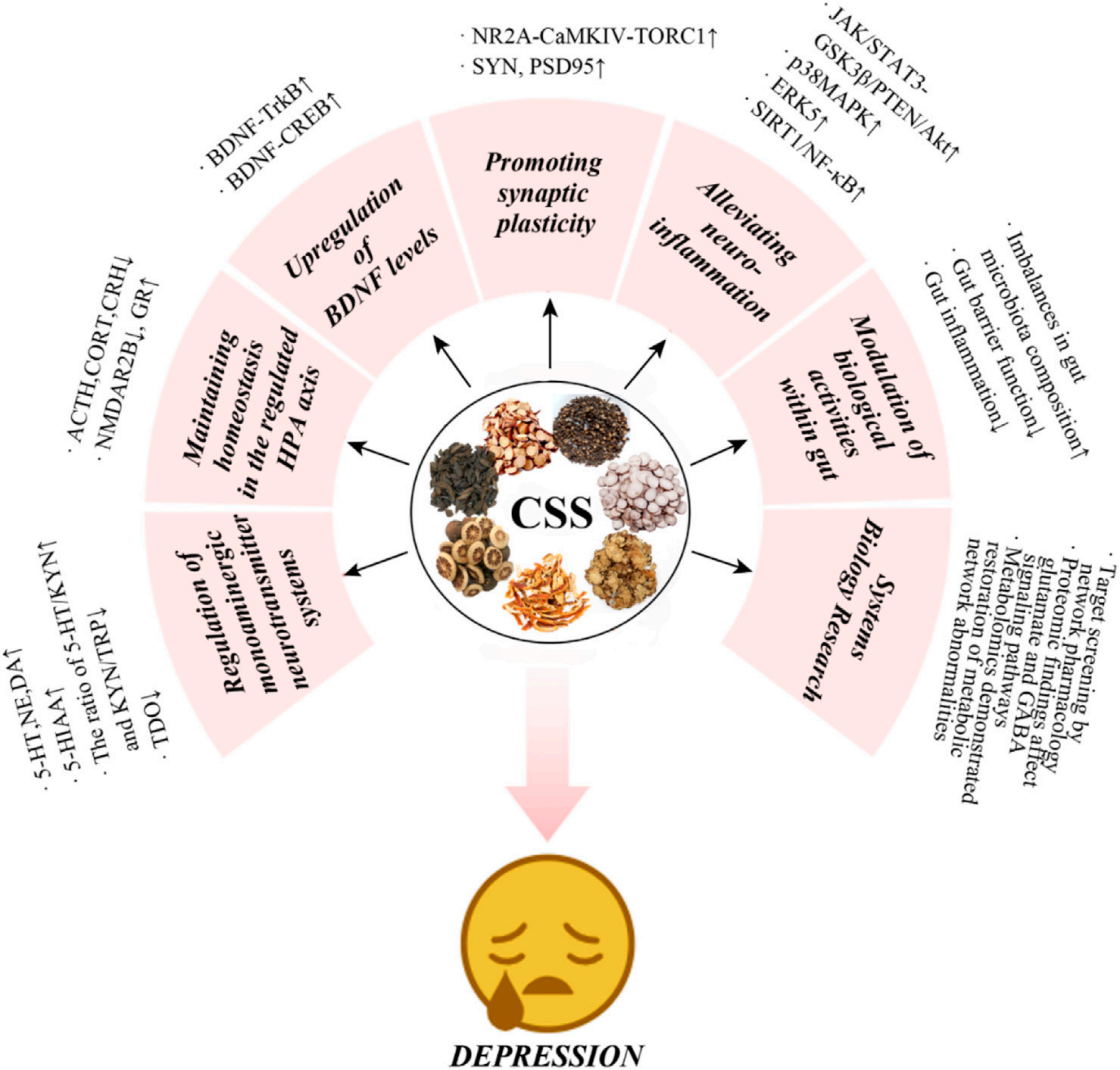
Multi-mechanistic effects of CSS in antidepressant therapy.

In summary, antidepressant effects of CSS are believed to be primarily related to monoamine neurotransmitters, the HPA axis, BDNF, synaptic plasticity, gut microbiota, and inflammatory responses (Figure 6; Table 2). It is thought to directly regulate these factors or indirectly act on various related proteins, receptors, or signaling pathways, which are primarily concentrated in the brain, the main targeted organ in current depression research.

**TABLE 2:** Research on the potential antidepressant-like effect of CSS.

Content	Dosage (kg/d)	Pharmaceutical manufacturing method	Model	Species	Treatment regimen	Positive control	Antidepression mechanisms	References
Bupleuri radix (19.05%), Citri reticulatae pericarpium (14.29%), Paeoniae radix alba (14.29%), Aurantii fructus (14.30%), Cyperi rhizoma (9.05%), Chuanxiong rhizoma (19.05%), Glycyrrhizae radix et rhizome (4.74%)	1.8 mg	Water extract	CUMS	SD rats	3w	Fluoxetine	Downregulate miR-124 expression and by releasing the inhibition of the MAPK14 and Gria3 signaling pathways	(Liu et al.,.2018)
Bupleuri radix (19.05%), Citri reticulatae pericarpium (19.05%), Paeoniae radix alba (14.29%), Aurantii fructus (14.29%), Cyperi rhizoma (14.29%), Chuanxiong rhizoma (14.29%), Glycyrrhizae radix et rhizome (4.76%)	20 mg	Water extract	CUMS	C57BL/6J mice	8w	Not Mentioned	Modulate BA metabolism by the gut microbiota-brain axis	Ma et al. (2022)
Bupleuri radix (19.05%), Citri reticulatae pericarpium (19.05%), Paeoniae radix alba (14.29%), Aurantii fructus (14.29%), Cyperi rhizoma (14.29%), Chuanxiong rhizoma (14.29%), Glycyrrhizae radix et rhizome (4.76%)	6 g	Water extract	CUMS	SD rats	2w	Escitalopram	Multiple targets and pathways, which may include regulations of 110 DEPs and some neurotransmitter’s transmission cycle	Zhu et al. (2023)
Bupleuri radix (13.85%), Citri reticulatae pericarpium (13.85%), Paeoniae radix alba (23.08%), Aurantii fructus (13.85%), Cyperi rhizoma (13.85%), Chuanxiong rhizoma (13.85%), Glycyrrhizae radix et rhizome (7.69%)	4 g	Freeze-drying	CRS	C57BL/6 mice	5 d	Buspirone	Induce NF-κB-involved BDNF expression through the regulation of gut inflammation and microbiota	Han et al. (2021)
Bupleuri radix (19.05%), Citri reticulatae pericarpium (19.05%), Paeoniae radix alba (14.29%), Aurantii fructus (14.29%), Cyperi rhizoma (14.29%), Chuanxiong rhizoma (14.29%), Glycyrrhizae radix et rhizome (4.75%)	1 g	Water extract	UCMS	SD rats	8w	Fluoxetine	Increase in the hippocampal ERα/ERβ mRNA ratio	Chen et al. (2020)
Bupleuri radix (13.85%), Citri reticulatae pericarpium (13.85%), Paeoniae radix alba (23.08%), Aurantii fructus (13.85%), Cyperi rhizoma (13.85%, Chuanxiong rhizoma (13.85%), Glycyrrhizae radix et rhizome (7.69%)	19.5 g	Water extract	CUMS	C57BL/6 mice	1w	Not Mentioned	Promote angiogenesis and neurogenesis in the hippocampus, targeting the SIRT1/FOXO1 axis and subsequent regulation of VEGFA and BDNF.	Zhang et al. (2023)
Bupleuri radix (19.05%), Citri reticulatae pericarpium (19.05%), Paeoniae radix alba (14.29%), Aurantii fructus (14.29%), Cyperi rhizoma (14.29%), Chuanxiong rhizoma (14.29%), Glycyrrhizae radix et rhizome (4.76%)	10 g	Water extract	OVX	SD rats	Not Mentioned	Not Mentioned	Regulate PI3k/AKT signaling pathway, liver and brain communication	Chen et al. (2021)
Radix Bupleuri (19.04%), Pericarpium Citri Reticulatae (19.04%), Rhizoma Chuan Xiong (14.29%), Rhizoma Cyperi (14.29%), Fructus Aurantii (14.29%), Radix Paeoniae Alba(14.29%), and Radix Glycyrrhizae (4.76%)	2.835	Water extract	CUMS	SD rats	4w	Fluoxetine	Suppress CHOP and caspase-12 mediated apoptosis in the rat hippocampus	Sun et al. (2020)
Bupleuri radix (19.05%), Citri reticulatae pericarpium (19.05%), Paeoniae radix alba (14.29%), Aurantii fructus (14.29%), Cyperi rhizoma (14.29%), Chuanxiong rhizoma (14.29%), Glycyrrhizae radix et rhizome (4.76%)	2.1	Water extract	CUMS	SD rats	4w	Fluoxetine	Regulate BDNF/ERK/CREB signaling pathway in the hippocampus and frontal cortex	Yan et al. (2020)
Bupleuri radix (19.05%), Citri reticulatae pericarpium (19.05%), Paeoniae radix alba (14.29%), Aurantii fructus (14.29%), Cyperi rhizoma (14.29%), Chuanxiong rhizoma (14.29%), Glycyrrhizae radix et rhizome (4.76%)	9.25/18.5	Water extract	CUMS	Wistar rats	6w	Fluoxetine	Regulate miR-155 expression in liver and brain, inhibiting liver-brain inflammation axis and TLR4/MyD88/NF-κB pathways	Ji et al. (2023)

## 5 Research on the antidepressant-like effects of the active ingredients of CSS

The ingredients detected in CSS using UPLC-QTOF-MS include ferulic acid, naringin, hesperidin, meranzin hydrate, glycyrrhizic acid, saikosaponin A, nobiletin, and hesperetin (Liu et al., 2020b). Additional ingredients such as ferulic acid, naringin, hesperidin, meranzin hydrate, glycyrrhizic acid, saikosaponin A, nobiletin, and hesperetin have also been identified in CSS.

HPLC analysis of CSS primarily identifies ingredients like naringin, neohesperidin, hesperidin, paeoniflorin, and glycyrrhetinic acid (Zhu et al., 2020b). LC-MS/MS analysis has revealed the presence of ingredients like paeoniflorin, ferulic acid, naringin, glycyrrhizic acid, saikosaponin A, chenpi flavonoid, and nobiletin in the water extract of CSS (Lei et al., 2021). These ingredients are considered the active ingredients responsible for the antidepressant-like effects of CSS.

In accordance with the 2020 edition of the Chinese Pharmacopoeia, the content of active ingredients in traditional Chinese medicine is regulated. For CSS, the minimum content of certain active ingredients is specified: Bupleuri radix should contain a total of not less than 0.30% of saikosaponin A (C42H68O13) and saikosaponin D (C42H68O13). Paeonia radix alba should contain not less than 1.2% of paeoniflorin (C23H28O11). Chuanxiong rhizoma should contain not less than 0.10% of ferulic acid (C10H10O4). Aurantii fructus should contain not less than 4.0% of naringin (C27H32O14). Citri reticulatae pericarpium should contain not less than 3.5% of hesperidin (C28H34O15). Cyperi rhizoma should contain not less than 1.0% of volatile oil, with Nootkatone as the characteristic ingredients. Glycyrrhizae radix et rhizome should contain not less than 0.50% of glycyrrhizin (C21H22O9) and not less than 2.0% of glycyrrhizic acid (C42H62O16). These standards ensure the quality and potency of CSS in traditional Chinese medicine. (They are listed in Table 3; Table 4).

**TABLE 3:** The active ingredients of CSS.

Source	Ingredients	Molecular formula	Structure	References
Bupleuri radix	Saikosaponin	C_42_H_68_O_13_	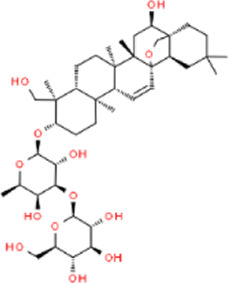	Liu et al. (2020b)
Paeonia lactiflora	Paeoniflorin	C_23_H_28_O_11_	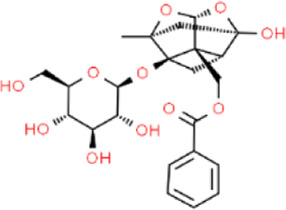	Lei et al. (2021)
Chuanxiong rhizoma	Ferulic acid	C_10_H_10_O_4_	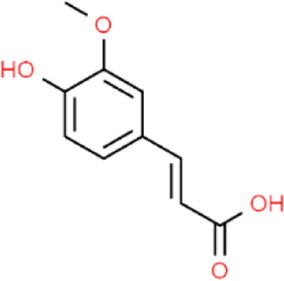	Liu et al. (2020b)
Aurantii fructus	Naringin	C_27_H_32_O_14_	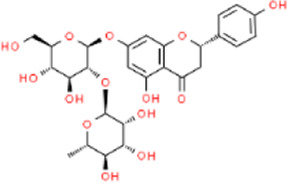	Su et al. (2014)
Citri reticulatae pericarpium	Hesperidin	C_28_H_34_O_15_	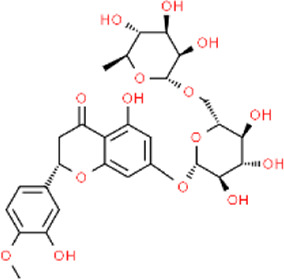	Liu et al. (2020b)
Cyperi rhizoma	Nootkatone	C_15_H_22_O	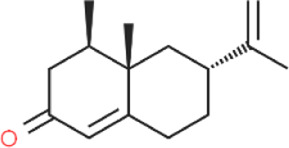	Wang et al. (2022)
Glycyrrhizae radix et rhizoma	Liquiritin	C_21_H_22_O_9_	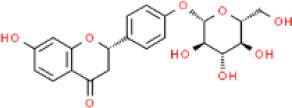	Zhu et al. (2020b)

**TABLE 4:** The antidepressant mechanisms of active ingredients of CSS.

Ingredient	Model	Species	Dose (mg/kg)	Positive control	Treatment regimen	Dissolving agent	Antidepressant mechanisms	References
Saikosaponin A	CSDS	C57BL/6 mice	25/50/100	Flu	Not Mentioned	Not Mentioned	Activate Tet1/DLL3/Notch signaling pathways	Tong et al. (2023)
	CUMS	SD rats	50	—	4w	NaCl	Increase levels of the hippocampal monoamine neurotransmitter DA	Guo et al. (2020)
	CUMS	Wistar rats	25/50/100	Flu	4w	NaCl	Rebalance the neuroendocrine system by alleviating neuroinflammation and restoring the neurotrophic system	Chen et al. (2018)
Saikosaponin D	CUMS	SD rats	0.75/1.5	Flu	3w	0.1% DMSO	Regulate Homer1-mGluR5 and mTOR pathway	Liu et al. (2022b)
	LPS	ICR mice	1	—	1w	0.1% DMSO	Inhibit neuroinflammation by downregulating HMGB1/TLR4/NF-κB signaling pathway	Su et al. (2020)
	CUMS	SD rats	0.75/1.5	Flu	3w	Not Mentioned	Negative regulation of NF-κB and thus downregulation of FGF2 expression by positive targeting of miR-155	Chao et al. (2020)
Paeoniflorin	CUMS	SD rats	30	U0126	5w	NaCl	Activate neuroprotection regulated by the ERK-CREB signaling pathway	Zhong et al. (2018)
	FST	SD rats	1	Flu	3 d	Not Mentioned	Regulating monoamine neurotransmitters, inhibit HPA axis hyperfunction and increase BDNF	Mu et al. (2020)
	LPS	SD rats	20/40/80	Flu	1w	Not Mentioned	Inhibit the activation of hippocampal microglia, activate FGF-2/FGFR1 signaling in neurons	Cheng et al. (2021)
Ferulic acid	TST	ICR mice	20/40/60	Flu	1w	0.1% methanol water	Enhance cell survival and proliferation, energy metabolism, and dopamine synthesis in mouse brain	Sasaki et al. (2019)
	CUS	C57BL/6 mice	40/80	Not Mentioned	3w	Not Mentioned	Regulate SIRT6/AKT/CRMP2 signaling pathway	Li et al. (2020b)
	PS	C57BL/6 mice	12.5/25/50	Flu	4w	0.5% CMC-Na	Inhibit proinflammatory cytokines and reorganize gut microbiome and microbial metabolism	Zheng et al. (2019)
Hesperidin	CUMS	SD rats	20/50/100	Flu	4w	Not Mentioned	Inhibit NLRP3 inflammasome and microglia activation	Xie et al. (2020b)
	CUMS	SD rats	50/98	Not Mentioned	70 d	1%	Activate Nrf2/ARE pathways for neuroprotection	Zhu et al. (2020a)
						CMC-Na		
Nootkatone	CUMS	C57BL/6 mice	6/12	Not Mentioned	4w	Not Mentioned	Inhibit NF-κB/NLRP3 pathways to mediate neuroinflammation	Yan et al. (2021)
	CUS	C57BL/6 mice	10	Not Mentioned	2w	0.9%	Increase neural regeneration in the hippocampal dentate gyrus, activate PKA/CREB pathways, and increase BDNF expression	Wang et al. (2022)
						NaCl		
Liquiritin	CUMS	ICR mice	10/20/40	Flu	1w	Not Mentioned	Inhibit microglia activation and release pro-inflammatory cytokine, protect hippocampal dendritic spine morphology	Chen et al. (2020)
	OVX	Wistar rats	20/40/80	Gennianan Tablelet	1w	Suspension	Influence the neuroendocrine-immune network	Lan et al. (2020)

### 5.1 Research on the antidepressant-like effects of saikosaponin

Saikosaponin possess a wide range of anti-inflammatory and immune-regulatory properties, making them effective ingredients in the treatment of depression with botanical drug medicine (Liu et al., 2020a; Wang et al., 2023b). The antidepressant effect of saikosaponin A may be associated with the modulation of DA levels and the transmembrane protein 2 rich in proline (PRRT2) in the hippocampus of depressive rats induced by CUMS (Guo et al., 2020). Saikosaponin A have been demonstrated to increase the expression of BDNF. In the MCAO + CUMS model, the administration of Chaihu Saponins significantly upregulated the protein levels of p-CREB, BDNF, Bax, and Caspase-3, promoting BDNF and Bcl-2 expression, consequently preventing hippocampal neuronal apoptosis and improving depressive-like behavior in post-stroke depression rats (Wang et al., 2021a).

As a critical regulator of the pro-inflammatory cytokine signaling pathway, NF-κB plays a pivotal role in immune responses, including stress, and has been associated with cognitive and memory impairments as well as depressive symptoms (Singh and Singh, 2020). Saikosaponin D can negatively regulate NF-κB, which, in turn, downregulates FGF2 expression through miR-155 downregulation. FGF2, as a key growth factor in synaptic plasticity and neuron growth, enhances synaptic plasticity and protects neurons, contributing to the antidepressant effect (Chao et al., 2020).

Saikosaponin A exhibits an antidepressant-like effect on peri-menopausal depressive rats induced by CUMS, and Saikosaponin A restores the hyperactivity of the HPA axis and pro-inflammatory cytokines while promoting BDNF-TrkB signaling in the hippocampus. SSA plays an antidepressant-like role in peri-menopausal rats by restoring hippocampal neuroendocrine, neuroinflammatory, and neurotrophic systems (Chen et al., 2018). Microglial cells serve as the first line of immune defense in the central nervous system, responding rapidly to external threats and secreting a substantial amount of oxidative stress substances and inflammatory cytokines, such as nitric oxide, reactive oxygen species, and IL-1β (Arioz et al., 2019). Activated microglial cells aim to remove antigens and restore brain homeostasis, but excessive activation can lead to neuronal damage. Saikosaponin D pretreatment effectively inhibits the release of inflammation-related cytokines (IL-1β, IL-6, and TNFα) mediated by activated microglial cells *in vitro* and *in vivo*. Saikosaponin D exerts its anti-inflammatory action by inhibiting the translocation and extracellular release of HMGB1, and inhibiting downstream TLR4/NF-κB signaling (Su et al., 2020). The elevated levels of glutamate induced by CUMS can lead to synaptic damage, which can be reversed through treatment with SSD. The amelioration of glutamate-induced synaptic injury appears to be mediated through the regulation of the Homer1-mGluR5 pathway and downstream mTOR signaling. It was observed that SSD treatment attenuated the increase in glutamate levels in the hippocampal CA1 region induced by chronic stress, which is known to contribute to synaptic damage and enhance the expression of synaptic proteins. Therefore, SSD may alleviate depressive-like behavior in CUMS-exposed rats by modulating the Homer1-mGluR5 and mTOR signaling pathways. These findings suggest that SSD can serve as a natural neuroprotective agent for the prevention of depression (Liu et al., 2022b). Therefore, saikosaponin A and saikosaponin D are promising ingredients in inhibiting the inflammatory response, which might be a potential mechanism underlying their antidepressant effects.

### 5.2 Research on the antidepressant-like effects of paeoniflorin

Paeoniflorin is a monoterpenoid glycoside obtained from the roots of peony or moutan, typically extracted from Bai Shao, a traditional low-toxicity Chinese botanical drug medicine widely used in the treatment of depression. Regardless of the type of stress animals are exposed to, reduced BDNF levels can potentially impact various brain functions, such as neurogenesis, neuron survival, and plasticity, promoting depressive-like behavior in stressed animals. Paeoniflorin exerts neuroprotective effects through the ERK-CREB signaling pathway, ameliorating depressive-like behavior in CUMS rats (Zhong et al., 2018). In an acute depression animal model induced by forced swimming, paeoniflorin significantly increases the serum and hippocampal BDNF levels in rats, offering protection against hippocampal pathological changes (Mu et al., 2020).

Oxidative stress is defined as an imbalance between the generation of reactive oxygen species (ROS) and the antioxidant capacity of cells, which is a major contributor to depression (Liu et al., 2023). Paeoniflorin can exhibit antidepressant effects through a multitarget pharmacological approach. For example, rats subjected to the forced swim test (FST) exhibit decreased plasma and hippocampal levels of serotonin, norepinephrine, and dopamine, as well as reduced plasma BDNF and superoxide dismutase (SOD) levels (Mu et al., 2020).

The HPA axis is associated with the pathophysiology of depression. Experimental evidence indicates that paeoniflorin can reverse depressive-like behavior. Neurogranin (Ng), a recently discovered postsynaptic protein widely distributed in the hippocampus, is found to be significantly lower in the hippocampus of depressive rats (Xiang et al., 2020). Paeoniflorin significantly increases the expression of Ng protein in the hippocampus. Furthermore, paeoniflorin decreases hippocampal glutamate (Glu) levels by inhibiting the expression of SNAP25, VAMP2, Syntaxin1a, and related proteins, as well as EAAT2/3, NR1, and NR2A proteins. These findings suggest that paeoniflorin can improve depressive-like behavior through modulation of the HPA axis, GR function, and Glu transporters (Li et al., 2020c).

Growing evidence suggests a close relationship between neuroinflammation and depression, making the inhibition of neuroinflammation a crucial treatment strategy for depression (Wei et al., 2023). Paeoniflorin can inhibit the TLR4/NF-κB/NLRP3 signaling pathway in the mouse hippocampus induced by LPS, reducing the levels of pro-inflammatory cytokines and microglial activation. Fibroblast growth factor-2 (FGF-2) plays a role in regulating neuron proliferation and differentiation (Koizumi, 2021). Paeoniflorin can increase FGF-2 levels and dendritic spine density. Therefore, paeoniflorin may exert its neuroprotective and antidepressant effects by inhibiting hippocampal microglial activation and activating the FGF-2/FGFR1 signaling pathway in neurons (Cheng et al., 2021).

### 5.3 Research on the antidepressant-like effects of ferulic acid

Ferulic acid (FA) is an aromatic acid originating from Chuanxiong (Szechuan lovage rhizome) and has shown antidepressant effects in clinical or animal studies (Zhang et al., 2018b). Among the various pharmacological actions of FA, its potential antidepressant effects have gained increasing attention. FA is a potent antioxidant that can scavenge hydrogen peroxide, hydroxyl radicals, superoxide radicals, and peroxynitrite (Xie et al., 2020a). Additionally, FA possesses characteristics of anti-apoptosis, anti-tumor, and anti-thrombotic properties. It can also inhibit neuroinflammatory responses and promote neurogenesis in the hippocampus (Kaur et al., 2022).

Hippocampal SIRT6 is involved in the *in vivo* lentivirus-mediated knockdown of depression induced by chronic unpredictable stress (CUS). Knockdown of hippocampal SIRT6 can prevent CUS-induced depressive-like phenotypes (Li et al., 2018). The PI3K/AKT signaling pathway is implicated in mediating antidepressant effects. The hippocampal SIRT6/AKT/CRMP2 signaling pathway is involved in the mechanism of antidepressant action of FA. CRMP2 is well-characterized as a target for neuronal plasticity and behavior regulation, regulating various aspects of neuron development, including axon guidance, dendritic morphology, and synaptic plasticity. Overexpression of CRMP2 effectively prevents acute axonal degeneration (Zhang et al., 2018a). CRMP2 activity is regulated by the SIRT6/AKT signaling pathway. Therefore, FA effectively activates the AKT/CRMP2 signaling pathway, improving CUS-induced depressive-like behavior and exerting neuroprotective effects through the SIRT6/AKT/CRMP2 pathway (Li et al., 2020b).

Ferulic acid can ameliorate depression in mice through antioxidative pathways. Studies have shown that ferulic acid increases the activities of SOD, catalase (CAT), and glutathione peroxidase (GSH-Px) in the blood, hippocampus, and cerebral cortex, while reducing levels of thiobarbituric acid reactive substances (TBA-RS). When the antioxidant defense system is modulated, ferulic acid can exert its antidepressant effects (Lenzi et al., 2015). In CUMS mice, FA improves depressive-like behavior in the tail suspension test (TST) and sucrose preference test (SPT). FA inhibits the activation of the NLRP3 inflammasome, the NF-κB signaling pathway, and the expression of IL-1β, IL-6, and TNF-α, suggesting that anti-inflammatory mechanisms are involved in antidepressant effects of ferulic acid in stressed mice (Liu et al., 2017).

Mitochondrial energy homeostasis mediated by ferulic acid is another potential mechanism of its antidepressant action. Dysfunction of mitochondria can lead to abnormal energy metabolism, triggering depressive episodes. Ferulic acid can promote glycogen metabolism in the brain, increase ATP levels, and activate neural function. FA also upregulates the gene expression of Ddc and Ppp1r1b, which are involved in dopamine signaling. FA treatment increases brain dopamine and norepinephrine levels, and thus, FA exerts its antidepressant effect by promoting dopamine synthesis (Sasaki et al., 2019).

Dysbiosis of the gut microbiota is closely associated with the onset of depression. FA effectively reduces the levels of pro-inflammatory cytokines, such as IL-6, IL-1β, TNF-α, while increasing the anti-inflammatory cytokine IL-10 in serum. This suggests that FA reduces systemic inflammation. 16S rRNA sequencing reveals that FA intervention reduces the abundance of the Proteobacteria phylum and significantly increases the abundance of *Lactobacillus* spp., Allobaculum spp., and Unspecified_S24_7. FA may exert its antidepressant effects by upregulating short-chain fatty acids. Furthermore, UHPLC-QTOF-MS/MS detection reveals that FA affects 459 microbial metabolites, primarily involved in tryptophan or thiamine metabolism. Tryptophan is an amino acid clinically used to alleviate depression, and a causal relationship exists between disrupted thiamine metabolism and depression (Wilson et al., 2023). Therefore, FA may exert its antidepressant effects by inhibiting pro-inflammatory cytokines, remodeling the gut microbiota, and modulating microbial metabolism (Hao et al., 2021).

### 5.4 Research on the antidepressant-like effects of naringin

Naringin is an active ingredient derived from the pericarp of citrus fruits, exhibiting a wide range of biological activities, such as anti-inflammatory and antioxidative properties (Kim and Kim, 2017), and has been employed in alleviating various neurological disorders (Emran et al., 2022). Adult hippocampal neurogenesis is crucial in the establishment of circuitry networks involving emotion-regulating regions like the amygdala and hypothalamus. Naringin promotes neuronal differentiation in normal mice, induces neural stem/progenitor cell (NSPC) migration to the subventricular zone-olfactory bulb (SVZ-OB) system, accelerates neuronal maturation in CORT-induced depressed mice, and enhances dendritic arborization, thereby exerting its antidepressant and anxiolytic effects via the promotion of neuronal differentiation target CREB (Gao et al., 2022). Moreover, naringin enhances BDNF through the cAMP-CREB-BDNF signaling pathway while suppressing neuroinflammation and neuronal apoptosis, ameliorating depressive behaviors in CORT-induced depressed mice (Zhang et al., 2023). Neurobehavioral characteristics of naringin are also accompanied by increased neuro-antioxidative and cholinergic activities. Furthermore, it significantly reduces the levels of malondialdehyde and nitrites, indicating involvement in the oxidative/nitrosative pathways, suggesting that naringin treatment may contribute to functional behavioral effects through mechanisms associated with enhanced cholinergic transmission, antioxidant defense systems, and the inhibition of lipid peroxidation and nitrosative processes (Ben-Azu et al., 2019). Rutin increases the levels of GAD67 in the striatum, prefrontal cortex, and hippocampus while reducing AChE activity. Additionally, Rutin region-dependently decreases TNF-α, IL-6, malondialdehyde, and nitrite concentrations while increasing glutathione levels. Our study suggests that Naringin attenuates SDS-induced depressive-like behaviors by increasing GAD67 synthesis, inhibiting AChE activity, oxidative and nitrosative stress, and neuroinflammation processes in stress-sensitive brain regions (Oladapo et al., 2021).

### 5.5 Research on the antidepressant-like effects of hesperidin

Hesperidin, an active ingredient derived from tangerine peel, possesses antioxidative, anti-inflammatory, and antiviral properties, while also promoting neurogenesis and enhancing memory function (Li et al., 2023). Studies have indicated that hesperidin exhibits antidepressant effects (Li et al., 2016). Hesperidin has been found to decrease the expression of the NLRP3 inflammasome (NLRP3, caspase-1, and ASC) in the prefrontal cortex (PFC) of rats induced with CUMS. It reduces the number of activated microglia in the PFC and levels of pro-inflammatory cytokines (IL-1β, IL-6, and TNF-α). Elevated levels of pro-inflammatory cytokines released by activated microglia contribute to neuroinflammatory responses, and hesperidin can inhibit the activation of pro-inflammatory cytokines in microglia induced by LPS and the NLRP3 signaling pathway (Xie et al., 2020b). In a comorbid diabetic depression rat model, hesperidin also exerts antidepressant effects. Hesperidin reduces depressive behaviors in diabetic rats by enhancing the function of Glo-1 in the amygdala and hippocampus, thereby reducing the formation of advanced glycation end products (AGEs) and oxidative stress-induced damage. Furthermore, the upregulation of Glo-1 by hesperidin is associated with the activation of the Nrf2/ARE signaling pathway, further elucidating the molecular mechanisms through which hesperidin inhibits the formation of brain AGEs and oxidative stress in diabetic rats. *In vitro* studies suggest that hesperidin treatment can reverse the reduced expression of Nrf2 induced by high glucose (HG). Therefore, the antidepressant-like effects of hesperidin are primarily mediated through the activation of the Nrf2/ARE/Glo-1 pathway (Zhu et al., 2020a). In a rat model of depression induced by PTSD, hesperidin can ameliorate depressive-like behavior in rats by inhibiting the activity of MAO-A and the expression of tryptophan hydroxylase-1 in the hippocampus. This leads to an increase in the release of serotonin (5-HT) (Lee et al., 2021).

### 5.6 Research on the antidepressant-like effects o research on the antidepressant-like effects of nootkatone

Nootkatone is the primary monomeric ingredient found in lotus plumule and exhibits various pharmacological properties, including antimicrobial, antioxidant, and anti-allergic activities. Nootkatone functions as a neuroprotective agent and improves cognitive impairments by inhibiting neuroinflammation through the TLR4/NF-κB/NLRP3 pathway in the hippocampus (Wang et al., 2018). Recent studies have shown that nootkatone also possesses antidepressant and anxiolytic effects by activating the Keap1/Nrf2/HO-1 antioxidant pathway in response to liver injury-induced depression (Yan et al., 2021). Nootkatone significantly increases the reduced expression of PKA and p-CREB caused by CUS, suggesting that the PKA/CREB pathway may be involved in regulating BDNF expression in response to nootkatone treatment. Additionally, Nootkatone improves neurogenesis in the hippocampal dentate gyrus, indicating that it may exert antidepressant effects by enhancing neurogenesis. Therefore, nootkatone likely exhibits antidepressant activity through increasing neurogenesis in the hippocampal dentate gyrus and activating the PKA/CREB pathway to enhance BDNF expression (Wang et al., 2022). In a CUMS-induced depression model, Nootkatone improves depressive symptoms by inhibiting neuroinflammation mediated by the NF-κB/NLRP3 pathway (Zhao et al., 2023a).

### 5.7 Research on the antidepressant-like effects of glycyrrhizin

Glycyrrhizin, the principal active ingredient in licorice, possesses pharmacological properties such as anti-inflammatory and antioxidant effects. A metabolomics analysis of the effects of Glycyrrhizin on depression in rats was conducted using UHPLC-Q-TOF MS and UHPLC-TQ MS, revealing the screening of 47 different metabolites in the urine of depressed rats. This suggests that Glycyrrhizin may exert its antidepressant effects through metabolic pathways (Yang et al., 2020). Glycyrrhizin can lower the levels of FGF-2 in the hippocampus of LPS-induced depressive model mice, increase dendritic spine density, and significantly reduce the number of Iba-1 positive cells. This indicates that Glycyrrhizin may exert its antidepressant effects by inhibiting the activation of microglial cells, suppressing the release of pro-inflammatory cytokines, and protecting dendritic spine morphology in the hippocampal region (Chen et al., 2020). Research also suggests that Glycyrrhizin exerts its antidepressant effects in part by inhibiting the levels of cytokines triggered by the NLRP3 inflammasome, highlighting the role of antioxidative stress and the inhibition of NLRP3 inflammasome activation in antidepressant properties of Glycyrrhizin (Liu et al., 2022a). Glycyrrhizin is applicable in various depression models, as it can reduce the levels of FSH and estradiol E2 in the serum, improve the decreased 5-HT and NE levels in the hypothalamus, and increase the levels of monoamine neurotransmitters, thereby ameliorating depressive-like behavior in perimenopausal rats (Lan et al., 2020).

## 6 Discussion

Extensive research has confirmed that the antidepressant mechanisms of CSS are primarily associated with the monoaminergic neurotransmitter system, the HPA axis, synaptic plasticity, BDNF, gut microbiota, neuroinflammation, among other factors. Additionally, the therapeutic effects of CSS extend to various brain regions, such as the hippocampus, prefrontal cortex, amygdala, and hypothalamus, as well as regulatory systems outside the central nervous system, including the liver, gastrointestinal tract, and endocrine system. Studies on the main active ingredients of CSS, such as saikosaponin, paeoniflorin, ferulic acid, and Nootkatone, have demonstrated their antidepressant effects. This suggests that antidepressant properties of CSS involve multiple targets, levels, and systems, making it a promising treatment for depression. However, the safety of the antidepressant mechanisms of CSS has not been fully elucidated. Therefore, it is crucial to identify and assess the quality of the effective ingredients within CSS.

While the efficacy of CSS as a holistic formula is well-established, the mechanistic explanations remain uncertain due to the limited comprehensive, systematic, and in-depth exploratory studies combining clinical trials, animal experiments, and cellular research. Further investigations using techniques like high-performance liquid chromatography and metabolomics are needed to explore which medicinal molecules can effectively cross the blood-brain barrier. Identifying the key molecules and understanding whether they exert antidepressant effects through gut microbiota or metabolic pathways is also an important area of research. Based on our current understanding and the limited scope of this paper, we have only discussed the mechanisms by which a subset of the identified active metabolites in CSS exert their effects. However, it is important to note that a botanical drug decoction may contain thousands of compounds, targeting multiple molecular pathways. Therefore, further identification of the effective ingredients in the decoction and their antidepressant effects remains a crucial area for future research (Xu et al., 2023).

## 7 Limitations and further perspective

While research on the mechanisms underlying the antidepressant effects of CSS and its active components has made certain advancements, there are inherent limitations in the existing studies. Firstly, most traditional Chinese medicine (TCM) compounds are characterized by intricate active ingredients, and previous research on the quality control of CSS lacks a standardized approach. This issue is not unique to CSS but is a common challenge in the study of various traditional botanical drug formulations. Discrepancies in the drug proportions of CSS have been observed in different studies, stemming from the clinical emphasis in TCM on individualized treatment based on specific patient conditions. However, such variations in drug composition adjustments are challenging to replicate in basic research. Therefore, it is imperative to establish reasonable research standards for prescription formulation in the course of basic research. Given the complexity of Chinese botanical drug ingredients, the mechanisms of action are not confined to a single component or target but involve numerous targets. Nevertheless, many experimental methods, primarily animal studies, tend to focus on revealing single pathways or individual target effects. Consequently, comprehensively and adequately exploring and elucidating the pharmacological mechanisms of CSS’s active components and the interactions between different mechanisms pose challenging tasks. In the future, TCM research could benefit from a multi-omics approach (metabolomics, transcriptomics, and proteomics) to extensively screen molecular mechanisms and potential targets for treating depression, leveraging the advantageous synergies of TCM in targeting multiple pathways, mechanisms, levels, and factors.

Secondly, studies on Chinese botanical drug medicine often underscore the absorption of relevant components into the bloodstream, their distribution in organs, metabolism, and excretion. However, the chemical composition of Chinese botanical drug medicine is highly intricate, and not all components may be absorbed into the bloodstream. Previous observations have noted that some botanical drug medicine components exhibit low bioavailability, with very low or undetectable concentrations in the blood. Enhancing the bioavailability of these components and focusing on constructing precise and efficient targeted systems are conducive to significantly improving treatment efficacy. However, there is limited research on enhancing the bioavailability of CSS in treating depression. Therefore, future studies should explore effective strategies for enhancing drug efficacy to provide clinical assistance, rather than merely validating its pharmacological mechanisms.

In practical clinical applications, Chinese botanical drug medicine is often a combination of prescribed herbs. However, current research predominantly concentrates on verifying the anti-depressive effects of individual components in the medication, deviating from traditional efficacy and prescription rules. Through extensive literature review and summarization, we propose a new research direction. Ferulic acid is identified as a crucial active component in CSS, with research indicating that its antidepressant effects at a dose of 50 mg/kg are similar to the overall prescription of CSS. Hence, exploring a novel combination approach by adding 50 mg/kg of ferulic acid to the overall CSS prescription may enhance the overall efficacy of CSS. This will be a focus of our future research.

It is essential to acknowledge that CSS lacks high-quality standardized clinical trials, including large-scale multicenter, randomized double-blind controlled trials, to validate its clinical efficacy in treating depression. Recognizing the involvement of multiple factors in the occurrence and development of diseases is crucial. For complex disorders like depression, a single etiology cannot fully explain its onset and progression. Therefore, the metabolism and transformation of CSS within the human body should be incorporated into future research. Integrated research utilizing various technologies and methods is necessary to comprehensively elucidate the antidepressant mechanisms of CSS, paving the way for new combination methods to increase drug effectiveness. This will establish a reliable scientific foundation for botanical drug antidepressant development and promote advancements in the field of TCM for treating depression.
